# PERCEIVED COGNITIVE FUNCTION: RELATIONSHIPS WITH DIABETES COMPLICATIONS AND SELF-EFFICACY IN LATINX OLDER ADULTS

**DOI:** 10.1093/geroni/igad104.2969

**Published:** 2023-12-21

**Authors:** Shenell Wood, Heather Cuevas

**Affiliations:** The University of Texas at Austin, Austin, Texas, United States; The University of Texas at Austin, Austin, Texas, United States

## Abstract

Cognitive dysfunction is a concern for individuals with type 2 diabetes (T2DM) and can affect diabetes self-management. However, it is often overlooked clinically and remains relatively understudied in Latinx older adults as a complication of T2DM. This project sought to explore the relationships between perceived cognitive dysfunction and its effects on self-efficacy and complications of T2DM in older Latinx adults. This was part of an analysis of baseline data from a larger randomized controlled trial investigating an cognitive rehabilitation intervention for older adults with T2DM. A total of 87 participants (mean age 67.3 +/- 5.4) completed a survey consisting of the Diabetes Empowerment Scale - short form (self-efficacy); PROMIS Bank v.2 - Cognitive Function (perceived cognitive function); and a self-report of number and type of diabetes complications. Participants had lower perceived cognitive dysfunction than the general population (Mean total score: 36.6 +/- 7.9); low self-efficacy (mean total score: 2.3 +/- 0.7) and reported at least 2 complications related to T2DM. Greater perceived cognitive dysfunction was related to lower self-efficacy (p = 0.04). Diabetes duration was positively associated with self-efficacy (p = 0.03), but not perceived cognitive dysfunction (p = 0.22). Self-efficacy was positively associated with number of diabetes complications (p = 0.03). Irrespective of diabetes duration, patients with more severe perceived cognitive dysfunction reported lower self-efficacy (p = 0.03) than those with lower levels of cognitive dysfunction. Assessment of cognitive function may help inform patient education interventions for Latinx older adults to improve the self-efficacy and potentially reduce complications.

